# Extrusion of the medial meniscus under a weight-loading condition in early knee osteoarthritis: an investigation using special upright magnetic resonance imaging

**DOI:** 10.1186/s12891-023-06807-x

**Published:** 2023-08-26

**Authors:** Kengo Shimozaki, Junsuke Nakase, Tomoyuki Kanayama, Yusuke Yanatori, Yoshihiro Ishida, Naoki Ohno, Tosiaki Miyati, Hiroyuki Tsuchiya

**Affiliations:** 1 Department of Orthopaedic Surgery, Graduate School of Medical Sciences, Kanazawa University, 13-1 Takara-Machi, Kanazawa, Ishikawa 920-8641 Japan; 2 Faculty of Health Sciences, Institute of Medical, Pharmaceutical and Health Sciences, Kanazawa University, Kanazawa, Ishikawa 920-0942 Japan

**Keywords:** Medial meniscus, Magnetic resonance imaging, Early knee osteoarthritis, Weight-loading condition

## Abstract

**Background:**

Whether the medial meniscus morphology and movement occur under upright loading conditions in early knee osteoarthritis (OA) or medial meniscus posterior root tear (MMPRT) remains unknown. This study aimed to evaluate the medial and anteroposterior extrusion of the medial meniscus under unloaded and upright-loaded conditions in patients with early knee OA.

**Methods:**

Twelve patients with early knee OA and 18 healthy adult volunteers participated in this study. Magnetic resonance imaging using special equipment was performed with the participants in the unloaded and upright-loaded conditions. Medial, anterior, and posterior extrusions of the medial meniscus against the tibial edge were evaluated and compared between the early knee OA and healthy adult control groups. Additionally, 12 patients in the early knee OA group were divided into 2 subgroups based on whether MMPRT was observed, and the extrusion of the medial meniscus was compared.

**Results:**

The amount of medial extrusion of the medial meniscus in both the unloaded and upright-loaded conditions was significantly greater in the early knee OA group than in the control group (unloaded: 2.6 ± 1.0 mm vs 0.7 ± 0.5 mm; upright-loaded: 3.7 ± 0.9 mm vs 1.8 ± 0.8 mm). Similarly, the anterior and posterior extrusion of the medial meniscus in the upright-loaded condition was significantly larger in the early knee OA group (anterior: 4.6 ± 1.0 mm vs 3.7 ± 1.1 mm; posterior: -3.4 ± 1.1 mm vs -4.6 ± 1.6 mm). However, no difference was observed in meniscal extrusion between unloaded and upright-loaded conditions. The posterior extrusion of the medial meniscus in the upright-loaded condition was significantly greater in MMPRT cases than in non-MMPRT cases in the early knee OA group (MMPRT: -2.7 ± 1.1 mm; non-MMPRT -4.1 ± 1.5 mm).

**Conclusions:**

In early knee OA, significantly large meniscal extrusions of the medial meniscus in both unloaded and upright-loaded conditions were found compared with healthy adults. Among patients with early knee OA, those with MMPRT showed a large posterior extrusion of the medial meniscus in the upright-loaded condition compared with those without MMPRT.

**Level of evidence:**

Level IV.

## Introduction

The incidence of knee osteoarthritis (OA), which leads to disabilities in daily life, is increasing in older people worldwide [1]. In Japan, there are over 8 million patients with knee OA, and many knee OA cases start from the medial compartment of the knee [2]. Given the progression of the knee OA, it may require surgical treatment, such as total knee arthroplasty or osteotomies around the knee; early knee OA does not present with the characteristic clinical symptoms or radiographic signs of established knee OA and has attracted scientific attention in recent years [3]. Although the etiology of early knee OA is not clear, some previous reports have shown an association between dysfunction of the medial meniscus due to meniscal degeneration and meniscal tears, such as medial meniscus posterior root tear (MMPRT) [4, 5]. As an indicator of meniscal dysfunction, medial extrusion of the medial meniscus is receiving attention and may lead to articular damage and progression of knee OA [6]. Although no clear standard value for abnormal medial extrusion of the medial meniscus has been established, large medial extrusion of the medial meniscus with or without meniscus tear is said to be a risk factor for the progression of knee OA [7–9].

However, assessments of meniscal extrusion are usually performed under unloaded conditions, and there are few reports on meniscal extrusion under weight-loading conditions. Recent studies have utilized custom-made loading magnetic resonance imaging (MRI) devices that measure parameters in the supine position. By using these MRI loading devices, meniscal extrusion and morphology during the loading condition of healthy knees [10], mild and moderate osteoarthritis [11], and meniscal tear cases [12] were investigated. Additionally, a previous report has shown that the medial meniscus moves medially owing to upright loading with little change in the anterior and posterior directions in healthy adults using upright weight-loading MRI [13]. Nevertheless, whether the medial meniscus morphology and movement occur under upright loading conditions in early knee OA or MMPRT cases remains unknown. Knowing the meniscus morphology and movement under loaded conditions in early knee OA may elucidate its pathology and develop some current treatment methods, such as lateral wedge insole [14], meniscus surgeries, and around-knee osteotomies [15, 16].

Therefore, this study aimed to evaluate the medial and anteroposterior extrusion of the medial meniscus between the unloaded and upright-loaded conditions in patients with early knee OA. The study hypothesis was as follows: the medial meniscus in patients with early knee OA will be extruded in the medial and posterior directions under loading conditions compared to that in healthy adults. Understanding meniscal extrusion of the medial meniscus under loading conditions in patients with early knee OA through this basic research is expected to provide clinically relevant anatomical information for orthopedic surgeons.

## Materials and methods

All procedures performed in this study involving human participants were in accordance with the ethical standards of the institutional and/or national research committee and with the 1964 Helsinki Declaration and its later amendments or comparable ethical standards. This retrospective study was approved by the ethics committee of our institution (approval no. 2328). Written informed consent for data publishing was obtained from all the patients and volunteers included in this study.

### Participants

Among patients with early knee OA who had medial knee pain with Kellgren–Lawrence (K–L) [17] grade 0 or 1 in a standing anteroposterior X-ray view between 2019 and 2020, 12 (4 men and 8 women) who underwent upright loading MRI for early knee OA were included in the early OA group. Early knee OA diagnosis was based on the following three criteria, similar to the study conducted by Luyten et al. [3]: Knee Injury and Osteoarthritis Outcome Score (KOOS) ≤ 85% in at least two of four categories, joint line tenderness or crepitus, and a K–L grade of 0 or 1. The other inclusion criteria for each group were as follows: no locking or catching findings suggestive of symptomatic or traumatic meniscal tear in the clinical examination; no history of ipsilateral knee surgery; no history of an obvious traumatic accident and ligament injury; no lateral pain in the knees or other parts; ability to undergo MRI assessment; absence of inflammatory diseases, as observed by MRI evaluation; and no pacemakers, body piercings, or tattoos. Furthermore, 18 healthy adults (13 men and 5 women) aged > 20 years with no history of knee pain or surgery volunteered to participate in the study as a control group.

A prior power analysis for the sample size was performed using G*Power software (latest ver. 3.1.9.7; Heinrich-Heine-Universität Düsseldorf, Düsseldorf, Germany), which revealed that for an effect size of 0.65, power of 0.6, and an α level of 0.05, 12 individuals were required in each group. The hip–knee–ankle (HKA) angle, absence and type of medial meniscus tear, medial and anteroposterior extrusion of the medial meniscus in unloaded and upright-loaded conditions, and changes in meniscal extrusion between unloaded and upright-loaded conditions were evaluated. In the early knee OA group, the HKA angle was measured using a standing anteroposterior whole-leg radiograph. By contrast, to avoid radiation exposure in young, healthy participants, digital photographs were used to assess the HKA angle in the control group [18].

### MRI

**Fig. 1:**
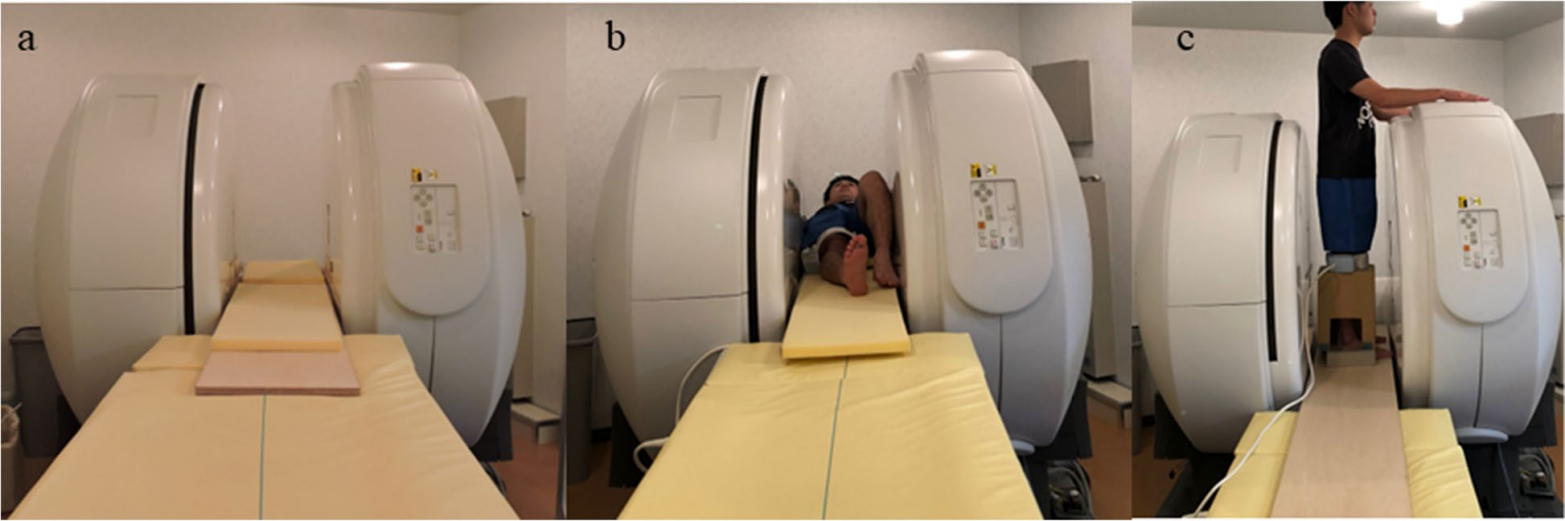
Process of magnetic resonance image capture: (**a**) MRI apparatus, (**b**) supine position, and (**c**) upright position. MRI, magnetic resonance imaging

All MRI studies were performed at our institution using a 0.4-T Gravity MRI system (Hitachi Healthcare, Tokyo, Japan) with a dedicated joint coil. The MRI apparatus was developed by improving the open-type MRI to extend its capability to capture images in any position, such as an upright loading position. In this study, the extrusions of the medial meniscus were measured in the unloaded and upright loaded positions (Fig. 1). Three-dimensional (3D) T1-weighted imaging of the knee was performed using pulse sequence, radiofrequency-spoiled steady-state gradient-echo at 29.6 ms of repetition time, 17.3 ms of echo time, 20° flip angle, 2-mm slice thickness, 256 × 192 imaging matrix, 512 × 512 reconstruction matrix, 220-mm field of view, one signal averaged, half-scan factor of 0.55, and 20 kHz receiver bandwidth. The knee was fixed in a fully extended position for each condition. T1-weighted images were obtained in coronal and sagittal planes. The imaging time for each condition was 3 min for each plane, with a 1-min break between each image.

**Fig. 2:**
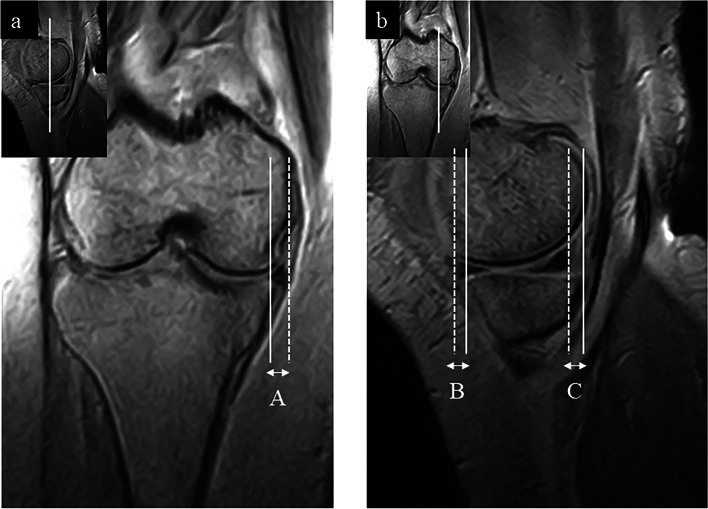
Evaluation of the meniscal extrusion on MRI. **a** Coronal plane evaluation image and (**b**) sagittal plane evaluation image. **A** Medial extrusion of the medial meniscus, (**B**) anterior extrusion, and (**C**) posterior extrusion of the medial meniscus. MRI, magnetic resonance imaging

After the MRI sequences were completed, image data were analyzed using a Synapse Vincent (Fuji Films, Tokyo, Japan). Medial, anterior, and posterior extrusions of the medial meniscus were assessed. Meniscal extrusion was defined as displacement from the margin of the tibial plateau. It was measured as the distance (mm) between the margin of the tibial plateau and peripheral border of the meniscal body. Therefore, if the meniscus did not protrude against the margin of the tibial plateau, it was indicated as a negative value. The image selected for the evaluation of the coronal plane included the central part of the anteroposterior diameter of the medial tibia in the sagittal plane, and the medial collateral ligament was best depicted on the coronal plane. The evaluation image for the sagittal plane included the middle of the medial femoral condyle on the coronal plane (Fig. 2). All measurements were performed on the unloaded and upright loading images.

An experienced orthopedic surgeon (K.S.) measured the extrusions of the medial meniscus three times at least 1 week apart in all cases. The mean outcomes of the three measurements were accepted as the results. The intra-class correlation coefficient (ICC) was calculated.

### Statistical analysis

Data were analyzed using IBM SPSS Statistics for Windows version 27.0 (IBM Corp., Armonk, NY, USA). The HKA angle, extrusion of the medial meniscus in both unloaded and upright loading conditions between the two groups, and changes in meniscal extrusion between the unloaded and upright loading conditions were compared using Welch’s t-test. Additionally, 12 patients in the early knee OA group were divided into 2 subgroups based on whether MMPRT was observed. The medial meniscus extrusion was compared between the subgroups using Welch’s t-test. The level of significance for all statistical analyses was set at α = 0.05.

## Results

The mean ages of the early knee OA and control group were 59.5 ± 7.5 and 21.8 ± 3.1 years old, respectively. The average HKA angles were 177.4° ± 3.4° in the healthy group and 177.0° ± 1.6° in the early knee OA group, which indicated mild varus of the knee, and no significant difference was found between the two groups (*P* = 0.73). Although no meniscal tears or degenerative changes were detected in the healthy group on MRI, six MMPRTs, four degenerative tears, and two non-particulars (six non-MMPRT cases) were recorded in the early knee OA group. The amounts of medial extrusion of the medial meniscus in both the unloaded and upright-loaded conditions were significantly greater in the early OA group than in the healthy group. Similarly, the anterior and posterior extrusion of the medial meniscus in the upright-loaded condition was significantly different between the healthy and early knee OA groups (Tables 1 and 2). No significant differences in the anterior and posterior extrusions of the medial meniscus in the unloaded condition and the change in meniscal extrusion between the unloaded and upright-loaded conditions were observed between the two groups (Table 3).

**Table 1:** Comparison of medial meniscal extrusion in the unloaded condition between the early knee OA and control groups

	Early OA (*n* = 12)	Control (*n* = 18)	*P* value
Medial extrusion (mm)	2.6 ± 1.0	0.7 ± 0.5	< 0.01
Anterior extrusion (mm)	4.6 ± 1.2	3.8 ± 1.0	0.06
Posterior extrusion (mm)	-3.9 ± 1.6	-4.7 ± 1.5	0.19

**Table 2:** Comparison of medial meniscal extrusion in the upright-loaded condition between the early knee OA and control groups

	Early OA (*n* = 12)	Control (*n* = 18)	*P* value
Medial extrusion (mm)	3.7 ± 0.9	1.8 ± 0.8	< 0.01
Anterior extrusion (mm)	4.6 ± 1.0	3.7 ± 1.1	0.04
Posterior extrusion (mm)	-3.4 ± 1.1	-4.6 ± 1.6	0.03

**Table 3:** Comparison of medial meniscal extrusion changes between the early knee OA and control groups

	Early OA (*n* = 12)	Control (*n* = 18)	*P* value
Change in medial extrusion (mm)	1.3 ± 0.5	1.2 ± 0.8	0.51
Change in anterior extrusion (mm)	0.0 ± 0.6	-0.1 ± 0.8	0.85
Change in posterior extrusion (mm)	0.4 ± 1.2	0.1 ± 1.0	0.81

In the early knee OA group, the posterior extrusion of the medial meniscus in the upright-loaded condition was significantly different between MMPRT and non-MMPRT cases (Table 5). In contrast, no significant differences between the medial and anterior extrusion of the medial meniscus in both the unloaded and upright-loaded conditions and change in medial meniscal extrusion were detected between the MMPRT and non-MMPRT cases (Tables 4, 5 and 6). For MRI assessment, the intra-class reliability ICC was 0.905.

**Table 4:** Comparison of medial meniscal extrusion in the unloaded condition between the MMPRT and non-MMPRT groups

	MMPRT (*n* = 6)	Non-MMPRT (*n* = 6)	*P* value
Medial extrusion (mm)	2.6 ± 0.3	2.3 ± 1.4	0.34
Anterior extrusion (mm)	4.5 ± 1.5	4.8 ± 1.0	0.65
Posterior extrusion (mm)	-3.5 ± 1.6	-4.3 ± 1.5	0.39

**Table 5:** Comparison of medial meniscal extrusion in the upright-loaded condition between the MMPRT and non-MMPRT groups

	MMPRT (*n* = 6)	Non-MMPRT (*n* = 6)	*P* value
Medial extrusion (mm)	3.9 ± 0.5	3.6 ± 1.3	0.50
Anterior extrusion (mm)	4.3 ± 1.2	4.7 ± 0.8	0.56
Posterior extrusion (mm)	-2.7 ± 1.1	-4.1 ± 1.5	0.04

**Table 6:** Comparison of medial meniscal extrusion changes between the MMPRT and non-MMPRT groups

	MMPRT (*n* = 6)	Non-MMPRT (*n* = 6)	*P* value
Change in medial extrusion (mm)	1.3 ± 0.4	1.2 ± 0.7	0.71
Change in anterior extrusion (mm)	-0.1 ± 0.6	-0.1 ± 0.6	0.96
Change in posterior extrusion (mm)	0.7 ± 1.4	0.2 ± 1.0	0.22

## Discussion

This study used an upright MRI apparatus to assess the extrusion of the medial meniscus under loading in patients with early knee OA, which has not been previously understood. The most crucial result of this study is that larger meniscal extrusions of the medial meniscus to the medial, anterior, and posterior in the upright-loaded condition were observed in patients with early knee OA than in healthy adults. Additionally, patients with early knee OA with MMPRT showed a large posterior extrusion of the medial meniscus in the upright-loaded condition, which may indicate that the loading stress on the posterior tibial plateau is large for knee extension and knee flexion under loading conditions.

Regarding the medial extrusion of the medial meniscus, the change in medial extrusion between unloaded and upright-loaded conditions was reported previously in healthy young and older adults using ultrasound and special MRI equipment. In young adults (aged 20–30 years), the medial extrusions of the medial meniscus were 0.8–0.9 mm in the unloaded condition and 1.4–1.8 mm in the upright-loaded condition [13, 19]. Conversely, the medial extrusions of the medial meniscus were 1.0–1.3 mm and 1.6–2.5 mm in the unloaded and upright-loaded conditions, respectively, in older adults (aged > 50 years) [10, 19]. In the early knee OA group of this study, the medial extrusions of the medial meniscus in both unloaded and upright-loaded conditions were larger than those in healthy young and older adults. One of the reasons for this result is the decreased quality of meniscal collagen tissue due to aging and meniscal tears due to meniscal degeneration [20]. As mentioned in the limitation section, bias owing to the presence of MMPRT cases is possible; however, even in cases without MMPRT, the medial extrusion of the medial meniscus was 2.3 mm in the unloaded and 3.6 mm in the upright-loaded condition, suggesting the involvement of meniscal degeneration. Another possible reason is that the meniscotibial ligament plays a critical role in connecting the meniscus and the tibia [21, 22]. A recent study pointed out that the medial extrusion of the medial meniscus in early knee OA is caused by dysfunction or damage to the meniscotibial ligament [23]. A detailed evaluation of the meniscotibial ligament was not possible in this study owing to the lack of MRI quality. However, further investigation of the meniscotibial ligament in early knee OA is required in the future. Although some previous studies have mentioned the anterior and posterior movements of the medial meniscus during knee flexion in healthy adults and those with early knee OA [4, 24], no reports have focused on meniscal extrusion under upright loading conditions with early knee OA. In healthy adults, no significant meniscal movement in the anteroposterior direction with upright loading is observed during knee extension [13]. Although a significant difference in anterior and posterior meniscal extrusions was observed between healthy subjects and patients with early knee OA under upright loading conditions, the amount of change because of loading was small, suggesting that the effect of meniscal degeneration and meniscotibial ligament damage was stronger than that of loading [20, 25].

In comparing MMPRT and non-MMPRT in patients with early knee OA, previous studies have shown that patients with MMPRT have more degeneration and greater posterior translation during knee flexion [26, 27]. The posterior movement of the medial meniscus leads to the progression of medial knee OA due to high contact pressure for the posterior cartilage, similar to that after a total meniscectomy [28]. Additionally, the results of the present study indicated that the posterior extrusion of the medial meniscus under upright loading during knee extension was larger in the MMPRT group than in the non-MMPRT group. Therefore, in treating MMPRT, both medial and posterior extrusion will possibly need to be treated [29].

Regarding the change in meniscal extrusion between unloaded and loaded conditions in this study, no difference was shown, as in previous reports [4, 30]. The change in the meniscal extrusion between unloaded and upright-loaded conditions was not different because the lower extremity alignment was also not different between the two groups, and there was not much increase of extrusion in MMPRT under loading condition, which was reported as the “dead meniscus sign” [30].

A clear strength of the current study was the use of upright MRI apparatus, which enabled examination during an upright weight-loading condition in early knee OA patients. This allowed us to evaluate for the first time the morphology of the medial meniscus during upright loading in patients with early OA, including MMPRT. Although every effort was made to ensure minimal limitations, the following limitations are acknowledged. First, the lack of high power owing to the small number of early knee OA case. We intend to plan future studies with larger numbers and adjusted background, such as age and body mass index. Second, the participants’ ages significantly differed between the two groups. This may have affected the meniscal extrusion results. Certainly, we would ideally need a healthy sample of the same age as the early knee OA group. However, the present results show a large extrusion compared with those of previous studies using healthy cases of the same age group [19]. There is no suspicion that the meniscus dynamics of early knee OA are different from normal. Third, half of the patients with early knee OA had MMPRT, which may have influenced the results. However, the amount of meniscal extrusion was not different between the MMPRT and non-MMPRT groups, except for posterior extrusion under upright loading conditions. We believe we have captured the characteristics of medial extrusion in patients with early knee OA. Further studies with more cases and adjusted backgrounds are warranted. Fourth, lower-limb alignment evaluation was performed using a digital camera rather than X-ray imaging in the healthy group. This was performed to avoid unnecessary radiation exposure in healthy young individuals. Finally, the MRI resolution was 0.4 T, which is unacceptable as 1.5 T and 3.0 T are often used currently. Therefore, an accurate evaluation of ligaments, such as the meniscotibial ligament, was impossible. However, the error in extrusion evaluation was small, and it was feasible to evaluate the meniscal extrusion of the medial meniscus in the present study.

Despite these limitations, as clinical relevance, understanding the morphology of the medial meniscus under upright loading conditions in patients with early knee OA is anticipated to improve surgeon knowledge and lead to further prevention and treatment studies for early knee OA.

## Conclusions

A significantly large medial extrusion of the medial meniscus in both unloaded and upright-loaded conditions and a significantly large anterior and posterior extrusion of the medial meniscus in the upright-loading condition were found in patients with early knee OA compared with healthy adults. Among patients with early knee OA, those with MMPRT showed a large posterior extrusion of the medial meniscus compared with those without MMPRT.

## Data Availability

The datasets used and/or analyzed during the current study are available from the corresponding author and first author on reasonable request.

## Abbreviations

OA: Osteoarthritis
MMPRT: Medial meniscus posterior root tear
MRI: Magnetic resonance imaging
K-L grade: Kellgren–Lawrence grade
HKA angle: Hip–knee–ankle angle
ICC: Intra-class correlation coefficient
